# Predicting major adverse cardiovascular events within 3 years by optimization of radiomics model derived from pericoronary adipose tissue on coronary computed tomography angiography: a case-control study

**DOI:** 10.1186/s12880-024-01295-4

**Published:** 2024-05-21

**Authors:** Rong-rong Zhang, Hong-rui You, Ya-yuan Geng, Xiao-gang Li, Yu Sun, Jie Hou, Lian-chang Ji, Jing-long Shi, Li-bo Zhang, Ben-qiang Yang

**Affiliations:** 1 Department of Radiology, General Hospital of Northern Theater Command, 83 Wenhua Road, Shenyang, Liaoning Province 110016 P.R. China; 2 Jinzhou Medical University, Jinzhou, China; 3 Shukun Technology Co., Ltd, West Beichen Road, Beijing, China; 4 Key Laboratory of Cardiovascular Imaging and Research of Liaoning Province, Shenyang, China

**Keywords:** Coronary computed tomography angiography, Pericoronary adipose tissue, Major adverse cardiovascular event, Radiomics

## Abstract

**Background:**

Coronary inflammation induces changes in pericoronary adipose tissue (PCAT) can be detected by coronary computed tomography angiography (CCTA). Our aim was to investigate whether different PCAT radiomics model based on CCTA could improve the prediction of major adverse cardiovascular events (MACE) within 3 years.

**Methods:**

This retrospective study included 141 consecutive patients with MACE and matched to patients with non-MACE (*n* = 141). Patients were randomly assigned into training and test datasets at a ratio of 8:2. After the robust radiomics features were selected by using the Spearman correlation analysis and the least absolute shrinkage and selection operator, radiomics models were built based on different machine learning algorithms. The clinical model was then calculated according to independent clinical risk factors. Finally, an overall model was established using the radiomics features and the clinical factors. Performance of the models was evaluated for discrimination degree, calibration degree, and clinical usefulness.

**Results:**

The diagnostic performance of the PCAT model was superior to that of the RCA-model, LAD-model, and LCX-model alone, with AUCs of 0.723, 0.675, 0.664, and 0.623, respectively. The overall model showed superior diagnostic performance than that of the PCAT-model and Cli-model, with AUCs of 0.797, 0.723, and 0.706, respectively. Calibration curve showed good fitness of the overall model, and decision curve analyze demonstrated that the model provides greater clinical benefit.

**Conclusion:**

The CCTA-based PCAT radiomics features of three major coronary arteries have the potential to be used as a predictor for MACE. The overall model incorporating the radiomics features and clinical factors offered significantly higher discrimination ability for MACE than using radiomics or clinical factors alone.

**Supplementary Information:**

The online version contains supplementary material available at 10.1186/s12880-024-01295-4.

## Introduction

Vascular inflammation is considered a crucial driver of atherogenesis and vulnerable plaque rupture, which results in subsequent adverse cardiac events [1–3]. There is growing interest in the detection of coronary artery inflammation, which has important implications for cardiovascular risk stratification and improving patient prognosis. For years, coronary computed tomography angiography (CCTA) has been used as a rule-out test for obstructive coronary artery disease (CAD) owing to its excellent negative predictive value [4–6], which substantial evidence suggests that CCTA-derived parameters provide important prognostic information, such as, lumen stenosis, plaque characteristics, plaque burden [7–9]. A recent study demonstrated that vascular wall inflammation-induced change in perivascular adipose tissue (PVAT) composition leads to an increase in CT attenuation, and this change can be captured by the perivascular Fat Attenuation Index (FAI) [10]. The level of inflammation captured by FAI is highly correlated with PET and can independently predict the progression of plaques and adverse cardiac events [10–12]. However, FAI is only based on voxel intensity values and does not consider the complex spatial relationship among voxels. Advanced radiomics analysis can overcome the deficiencies of FAI and reveal the microstructure and composition changes in the parenchyma of PCAT.

Radiomics is the process of extracting quantitative features from a given ROI, converting images into mineable data, and analyzing these data for decision support [13–15]. Recently study reported CCTA-based radiomics characterization of PCAT surrounding the right coronary artery (RCA) and identified a distinct radiomics phenotype between patients with acute myocardial infarction (MI) and matched subjects with stable CAD or no CAD [16]. However, the role of the radiomics phenotype derived from PCAT surrounding the left anterior descending artery (LAD) and left circumflex coronary artery (LCX) was not mentioned, and whether the predictive value of PCAT radiomics features surrounding the three coronary arteries can be improved remains uncertain. Therefore, in this study, we sought to analyze CCTA-based radiomics features of PCAT surrounding the proximal three major coronary arteries. We aimed to predict future adverse cardiac events within 3 years using radiomics signature of PCAT and incorporating clinical risk factors.

## Materials and methods

### Study population

This retrospective study was approved by the institutional review board (No. Y [2021] 074) of the General Hospital of Northern Theater Command, and a waiver for informed consent was granted.

**Fig. 1:**
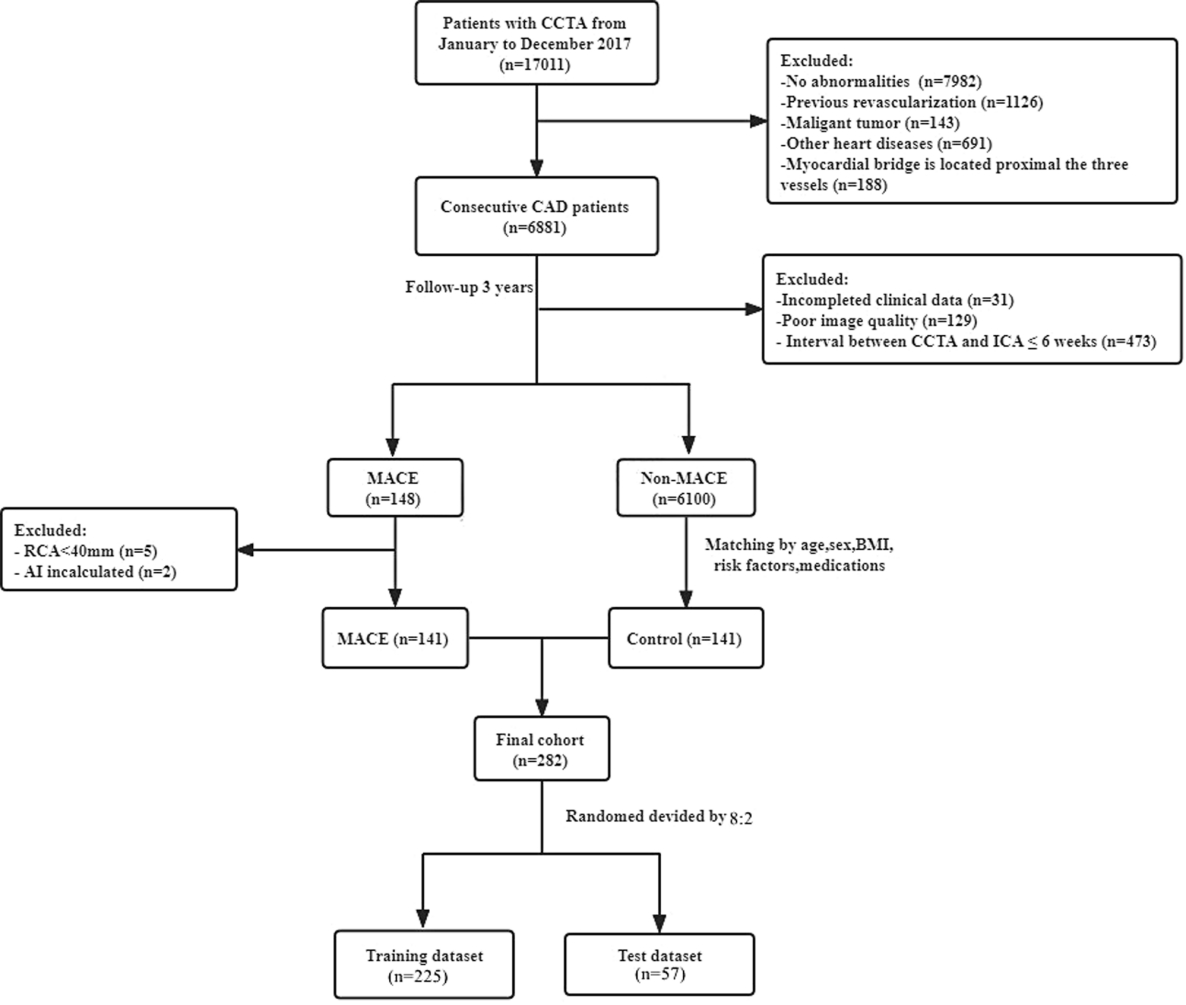
The Flow chart of patient enrollment

Our study was a post hoc retrospective analysis of consecutive patients who underwent 256-slice CCTA examinations from January 2017 to December 2017 and followed up until March 2021. Clinical outcome was defined as the occurrence of MACE based on electronic medical records of the hospital. MACE was defined as cardiac death (fatal MI), non-fatal MI (ST-segment elevation myocardial infarction [STEMI] and non-STEMI), or unstable angina leading to coronary revascularization (percutaneous coronary intervention [PCI] or coronary artery bypass grafting [CABG]) with more than 6 weeks between CCTA and invasive coronary angiography (ICA) [17]. Patients with MACE were matched (according to age, sex, body mass index (BMI), cardiovascular risk factors, and medications) to non-MACE patients who underwent CCTA during the same period. Exclusion criteria included previous coronary revascularization, coronary revascularization within 6 weeks of the CCTA scan, myocardial bridge is located proximal the three major vessels, patients with other heart diseases, poor CCTA image quality, and patients with a malignant tumor. A flowchart of patient selection is provided in Fig. 1. Relevant clinical information, laboratory parameters, and medications were retrospectively collected from the hospital’s electronic medical record system at the same time CCTA scans.

### CCTA acquisition

CCTA scans were performed on a 256-slice CT scanner (Brilliance iCT, Philips Medical systems). Sublingual nitroglycerin was used to dilatate coronary arteries, and oral or intravenous beta-blockers was administered as needed to reduce heart rate to ≤ 65 beats/min unless contraindicated. CCTA acquisition parameters were as follows: collimation was 128 × 0.625 mm, rotation time was 270 ms, tube voltage was set at 100 or 120 kV (depending on BMI), and the tube current was 500–700 mAs. Data were acquired using a retrospective electrocardiogram-gated protocol. 0.6–0.8 ml/kg of iodinated contrast was injected (Ioversol 320mgI/ml) at a flow rate of 4–6 ml/s, and images were reconstructed at a window centered at 75% of the R-R interval with a section thickness of 0.9 mm and a reconstruction increment of 0.45 mm.

### PCAT analysis and radiomics feature extraction

PCAT segmentation and radiomics feature extraction were performed using the Perivascular Fat Analysis Tool software (Shukun Technology Co., Ltd). The proximal 40-mm segments of the three major epicardial coronary arteries (LAD, LCX, and RCA) were automatically traced. For the LAD and LCX, we analyzed the proximal 40 mm from the left main coronary artery bifurcation. To avoid the effects of the aortic wall, we excluded the most proximal 10 mm of the RCA and analyzed the proximal 10–50 mm of the vessel, as described previously [10]. The PCAT was defined as all voxels located within the outer radial distance from the coronary wall equal to the diameter of the respective vessel, with CT attenuation between − 190 and − 30 HU [10, 11].

**Fig. 2:**
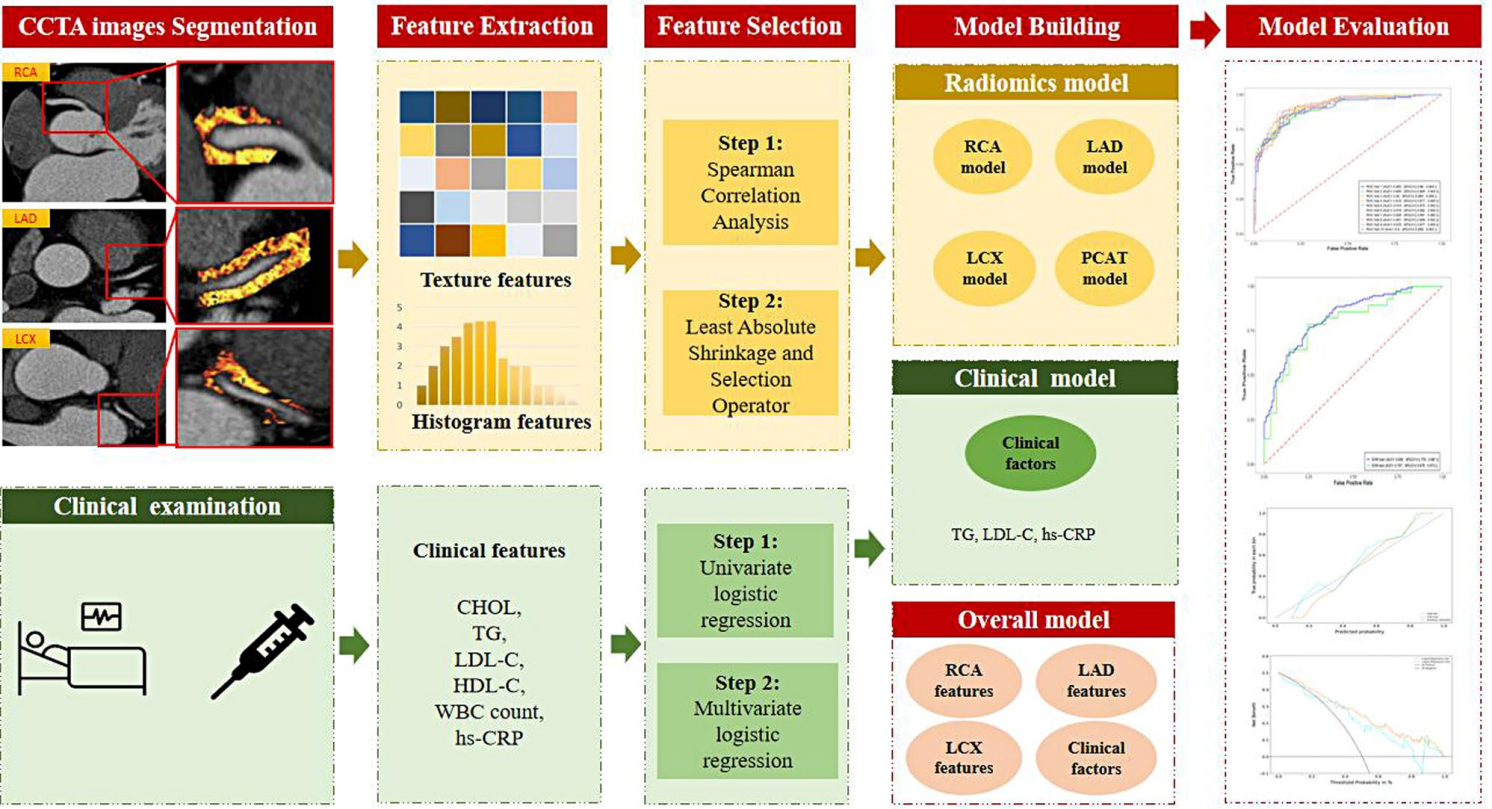
Radiomics workflow in this study

A total of 95 features (Supplementary Materials) were extracted from each PCAT segmentation. Finally, 285 radiomics features were generated from each patient. Figure 2 shows the radiomics workflow of this study.

## Feature selection and model building

### Feature selection

All cases in the training dataset were used to train the predictive model, whereas cases in the test dataset were used to independently evaluate the model’s performance. Before feature selection, variables with zero variance were excluded from analyses, and data were standardized by z-score. The calculation formula was as follows:

$$\text{F}=\frac{Fi-Mean}{\sigma },$$

where F signifies the normalized eigenvalue, Fi signifies the original eigenvalue, Mean signifies the average, and σ signifies the standard deviation (SD).

To reduce redundancy, features with a Spearman’s correlation coefficient of > 0.9 were excluded. The Least Absolute Shrinkage and Selection Operator (LASSO) was then used to select the robust features in the training group. Clinical features related to MACE were evaluated using univariate logistic regression analysis in the training dataset. Variables with *P* < 0.05 were included for further multivariate logistic regression analysis using the backward stepwise elimination method.

### Model building

A total of six models were developed using different machine learning algorithms (logistic regression (LR), support vector machine (SVM), stochastic gradient descent (SGD), and linearSVC). To avoid overfitting, all models were evaluated by 10-fold cross-validation.

#### Clinical model building

We developed a clinical model (Cli-model) based on the independent clinical risk factors selected using univariate and multivariate logistic regression analyses.

#### Radiomics model building

A total of four radiomics models were established. Firstly, three separate models were built based on the radiomics features selected from the PCAT surrounding the RCA, LAD, and LCX, respectively. Secondly, a PCAT model containing all features of RCA, LAD and LCX was constructed.

#### Overall model building

Finally, an overall model that incorporated the clinical risk factors and the radiomics features of three coronary arteries was established.

## Performance evaluation

We evaluated the performance of the models in terms of discriminative degree, calibration degree, and clinical usefulness.

### Discriminative degree

To evaluate the discriminative ability of the models, ROC curves were plotted for both the training and test datasets. AUCs with 95% CIs were calculated and compared using the DeLong test. The optimum cut-off value was determined by the maximum Youdon index of the training dataset, which was then applied to the independent test dataset. Sensitivity, specificity, positive predictive value (PPV), negative predict value (NPV), and accuracy were calculated according to the best cut-off value for both the training and test datasets.

### Calibration degree

Calibration curves using the Hosmer-Lemeshow test (H-L test) were generated to assess the goodness of fit of both the training and test datasets. The agreement between the observed outcome frequencies and the predicted probabilities of the models was assessed. A *P*-value of more than 0.05 in the H-L test was considered good calibration.

### Clinical usefulness

Decision curve analysis (DCA) was conducted to assess the clinical usefulness of the established models by quantifying the net benefit at different threshold probabilities of both the training and test datasets.

### Statistical analysis

All statistical analyses were conducted using the R Studio software. The Kolmogorov–Smirnov test was used to evaluate whether the continuous variables were normally distributed. Continuous variables are presented as means ± SDs or medians (interquartile ranges [IQRs]), as appropriate. Student’s *t*-test or Mann–Whitney *U-*test was used to compare differences in the continuous variables between groups, as appropriate. Categorical variables are presented as frequencies (percentages) and were compared using the chi-squared or Fisher’s exact test, as appropriate. All statistical analyses were two-sided, and *P* < 0.05 indicated statistical significance.

## Results

### Patients

Table 1 shows the clinical characteristics of the training and test datasets. The two groups were well matched for age, sex, BMI, cardiovascular risk factors, and medications in both the training and test datasets (all *P* > 0.05). The average time from the CCTA examination to the occurrence of MACE was 13.17 months (IQR: 4.9 to 29.17 months). In those who had experienced a MACE, 26 (18%) subjects experienced MI, 112 (80%) subjects with unstable angina underwent late revascularization (103 PCI and 9 CABG), and 3 (2%) patients had cardiac death (Table 2). In terms of lipids and inflammatory markers, the univariate analysis found that total cholesterol (CHOL), triglycerides (TG), low-density lipoprotein (LDL-C), and high-sensitivity C-reactive protein (hs-CRP) were significantly associated with MACE in the training dataset (*P* < 0.05).

**Table 1:** Baseline characteristics of patients in the training and test datasets

Variable	Training					Test				*P* value
	Control(n = 112)	MACE(n = 113)	Statistics	P-value		Control(n = 29)	MACE(n = 28)	Statistics	P-value	
Demographic features										
Male	70 (62.50%)	69 (61.10%)	0.049	0.824		17 (58.60%)	18 (64.30%)	0.193	0.661	0.959
Age, years	60.50 ± 9.31	59.97 ± 9.07	0.430	0.830		57.76 ± 9.22	59.82 ± 10.70	0.597	0.597	0.588
BMI, kg/m^2^	26.00(24.00, 28.00)	26.00(23.00, 27.00)	-0.747	0.455		25.00(23.50, 27.00)	25.00(24.00, 27.00)	-0.314	0.754	0.211
Cardiovascular risk factors										
Hypertension	78 (69.60%)	78 (69.00%)	0.010	0.920		22 (75.90%)	23 (82.10%)	0.338	0.561	0.152
Hyperlipidemia	12 (10.70%)	11 (9.70%)	0.059	0.808		2 (6.90%)	3 (10.70%)	0.259	0.967	0.744
Diabetes	43 (38.40%)	47 (41.60%)	0.240	0.624		14 (48.30%)	7 (25.00%)	3.317	0.069	0.663
Smoking	38 (33.90%)	37 (32.70%)	0.036	0.850		6 (20.7%)	14 (50.00%)	5.373	0.151	0.802
Medications										
ß-blocker	4 (3.60%)	7 (6.20%)	0.832	0.362		2 (6.90%)	3 (10.70%)	0.002	0.967	0.258
ACE-I/ARB1	28 (25.00%)	29 (25.70%)	0.013	0.909		9 (31.10%)	5 (17.90%)	1.335	0.248	0.905
Ca2+	33 (29.50%)	31 (27.40%)	0.114	0.736		7 (24.10%)	16 (57.10%)	6.447	0.011	0.082
Statin	12 (10.70%)	20 (17.70%)	1.077	0.134		8 (27.60%)	5 (17.90%)	0.766	0.381	0.300
Antiplatelet	12 (11.43%)	14 (13.33%)	2.249	0.675		6 (20.70%)	4 (14.30%)	0.404	0.525	0.529
Lipids, mmol/l										
CHOL	4.26 (2.74,4.93)	4.81 (3.82,5.68)	-4.397	< 0.001*		4.46 (3.63,5.19)	5.72 (4.31,6.84)	-3.129	0.002*	0.021*
TG	1.40 (0.97,1.89)	1.39 (1.04,2.46)	-2.116	0.034*		1.26 (1.03,1.67)	2.14 (1.71,3.84)	-4.302	< 0.001*	0.010*
HDL-C	1.05 (0.91, 1.25)	1.08 (0.88, 1.40)	-0.980	0.327		1.15 (0.85,1.38)	1.06 (0.88, 1.35)	-0.391	0.696	0.629
LDL-C	2.36 (1.38,2.83)	2.81 (2.07,3.63)	-4.999	< 0.001*		2.73 (2.01,3.10)	3.24 (2.34,4.14)	-2.259	0.024*	0.041
Inflammatory markers										
WBC count, *10^9^/l	6.63 (5.70, 7.90)	7.20 (5.65, 8.40)	-1.396	0.163		6.20 (5.10, 7.86)	7.35 (6.00, 8.38)	-3.162	0.002*	0.075
hs-CRP, mg/l	2.60 (0.90, 4.70)	3.60 (2.50, 4.65)	-2.277	0.023*		1.80 (0.70, 3.51)	3.70 (2.75, 4.57)	-2.172	0.030*	0.464

*MACE* major adverse cardiac event, *BMI* body mass index, *ACE-I* angiotensin converting enzyme inhibitor, *ARB* angiotensin receptor blocker, *Ca2 +* calcium channel blocker, *CHOL* total cholesterol, *TG* triglycerides, *HDL-C* high-density lipoprotein cholesterol, *LDL* low-density lipoprotein cholesterol, *WBC* white blood cell, *hs-CRP* high-sensitivity C-reactive protein, *UA* unstable angina

*indicated *P* < 0.05 with significance

**Table 2:** The exact events of MACEs in the training and test datasets

MACE events, n (%)	Total (n = 141)	Training dataset (n = 113)	Test dataset (n = 28)	X^2^	*P*
UA	112 (79.4)	93 (82.3)	19 (67.9)	2.865	0.090
MI	26 (18.4)	18 (15.9)	8 (28.6)	2.385	0.123
Death	3 (2.1)	2 (1.8)	1 (3.6)	0.000	1.000

*OR* odds ratio, *CI* confidence interval, *LDL-C* low-density lipoprotein cholesterol, *WBC* white blood cell, *TG* triglycerides

## Feature selection and modeling

### Clinical factor selection and clinical model building

Multivariate logistic regression analysis of the clinical characteristics revealed that LDL-C, hs-CRP, and TG were significant predictors of MACE (*P* < 0.05) in the training dataset. The Cli-model was established based on selected risk factors.

### Radiomics feature selection and radiomics model building

After removing the redundant features using Spearman’s correlation analysis, 38 features from the RCA, 34 features from the LAD, 34 features from the LCX, and 31 features from the PCAT remained and were identified by the LASSO algorithm to select the strongest features with significant value for predicting MACE. After the LASSO analysis, 15, 9, 7, and 15 features remained (Supplementary Materials) for RCA, LAD, LCX, and PCAT respectively, to construct their respective models.

### Overall model building

Finally, the selected clinical risk factors and the radiomics features of the three coronary adipose tissue (RCA, LAD, and LCX) were incorporated into an overall model.

## Performance evaluation

### Discriminative degree

**Fig. 3:**
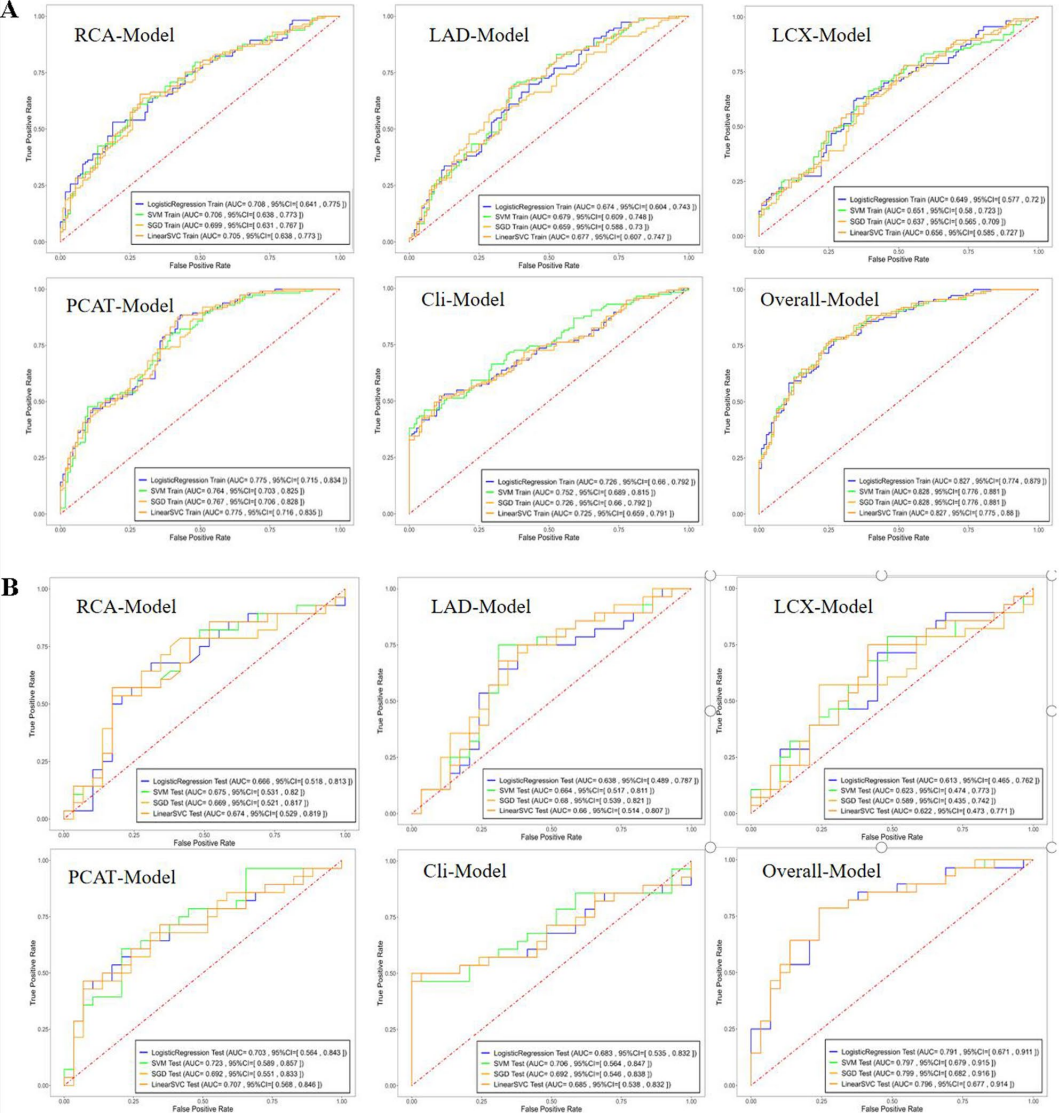
The ROC curves of the machine learning algorithms with different models in the training (**A**) and test dataset (**B**)

ROC curves for each model and ML algorithms are shown in the training (Fig. 3a) and test (Fig. 3b) datasets (Supplementary Table S2). The radiomics model constructed using the SVM method demonstrated the best performance, thus we compared the prediction ability of different models based on the SVM method.

**Fig. 4:**
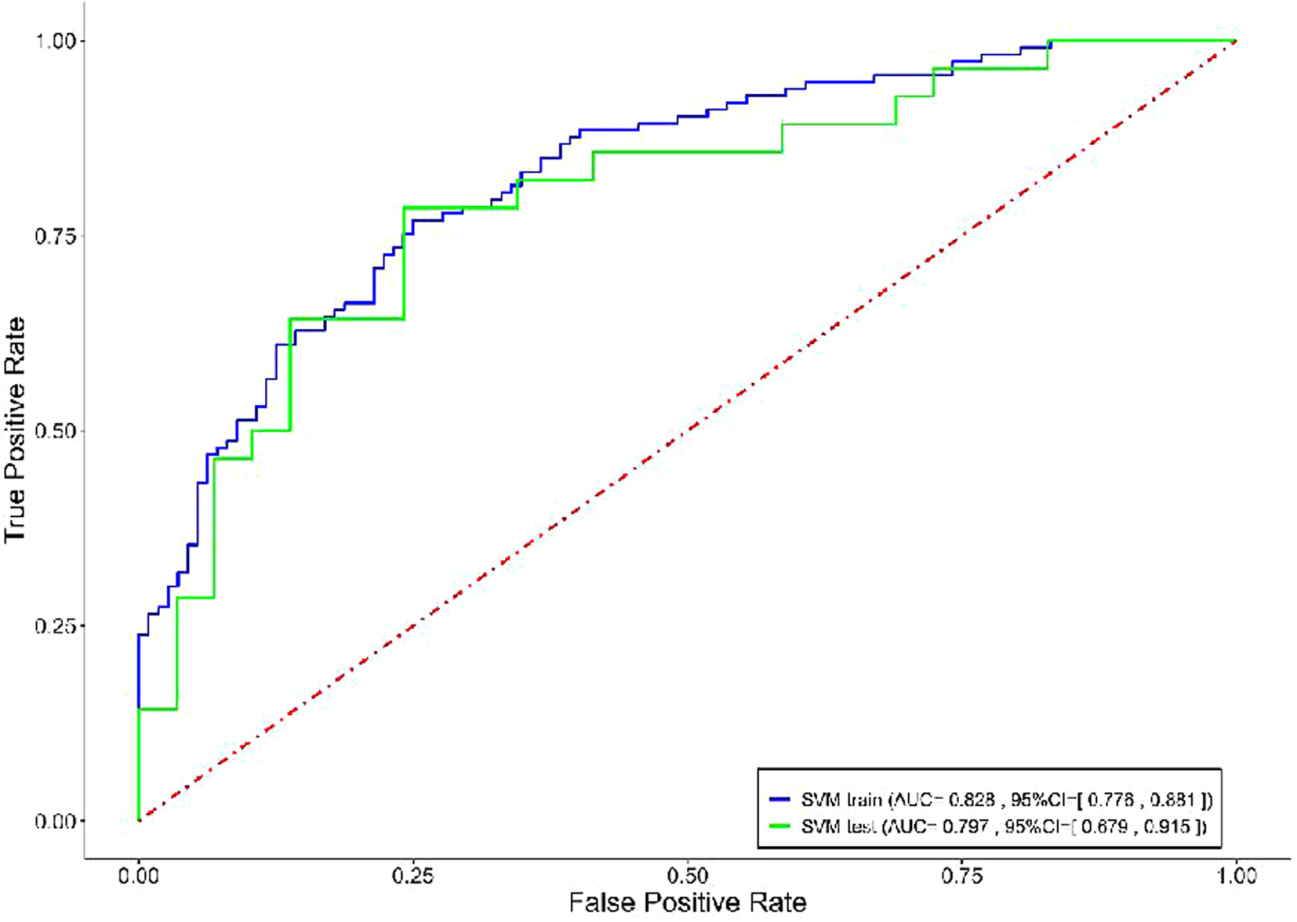
The ROC curves of overall model for predicting MACE in the training and test dataset

The Cli-model had AUCs of 0.752 and 0.706 in the training and test datasets, respectively. The PCAT-model, which integrated the RCA, LAD, and LCX features exhibited superior predictive performance than that of the RCA-model, LAD-model, and LCX-model alone with AUCs of 0.764 and 0.723 in the training and test datasets, respectively (Table 3). The RCA-model alone performed better than both the LAD-model alone and LCX-model alone, with AUCs of 0.706, 0.679, and 0.651, respectively, in the training dataset and 0.675, 0.664, and 0.623, respectively, in the test dataset. And the stratified analysis showed the scores of three major coronary vessels were not affected by the tube voltage (Supplementary Table S3). The overall model improved significantly after incorporating the clinical factors and radiomics features, with AUCs of 0.828 and 0.797 in the training and test datasets, respectively. ROC curves of both the training and test datasets are shown in Fig. 4. The accuracy, sensitivity, specificity, PPV, and NPV of each model are summarized in Table 3. The DeLong test showed that the AUCs of the training and test groups were not significantly different, with *P >* 0.05 for the Cli-model, PCAT-model, and overall model.

**Table 3:** The performance of the models in the training and test datasets

Model	Accuracy			AUC (95%CI)			Sensitivity			Specificity			PPV			NPV	
	Train	Test		Train	Test		Train	Test		Train	Test		Train	Test		Train	Test
LAD-Model	0.662	0.649		0.679 (0.609, 0.748)	0.664 (0.517, 0.811)		0.699	0.785		0.625	0.517		0.653	0.611		0.673	0.714
LCX-Model	0.636	0.614		0.651 (0.580, 0.723)	0.623 (0.474, 0.773)		0.664	0.643		0.607	0.586		0.630	0.600		0.642	0.630
RCA-Model	0.662	0.684		0.706 (0.638, 0.773)	0.675 (0.531, 0.820)		0.584	0.571		0.741	0.793		0.695	0.727		0.639	0.657
PCAT-Model	0.702	0.649		0.764 (0.703, 0.825)	0.723 (0.589, 0.857)		0.797	0.714		0.607	0.586		0.672	0.625		0.747	0.680
Cli-Model	0.702	0.667		0.752 (0.689, 0.815)	0.706 (0.564, 0.847)		0.460	0.464		0.946	0.862		0.897	0.765		0.635	0.625
Overall Model	0.760	0.719		0.828 (0.776, 0.881)	0.797 (0.679, 0.915)		0.770	0.643		0.750	0.793		0.757	0.750		0.764	0.697

*LAD* left anterior descending artery, *LCX* left circumflex coronary artery, *RCA* right coronary artery, *AUC* area under the curve, *CI* confidence interval, *PPV* positive predict value, *NPV* negative predict value

### Calibration degree

**Fig. 5:**
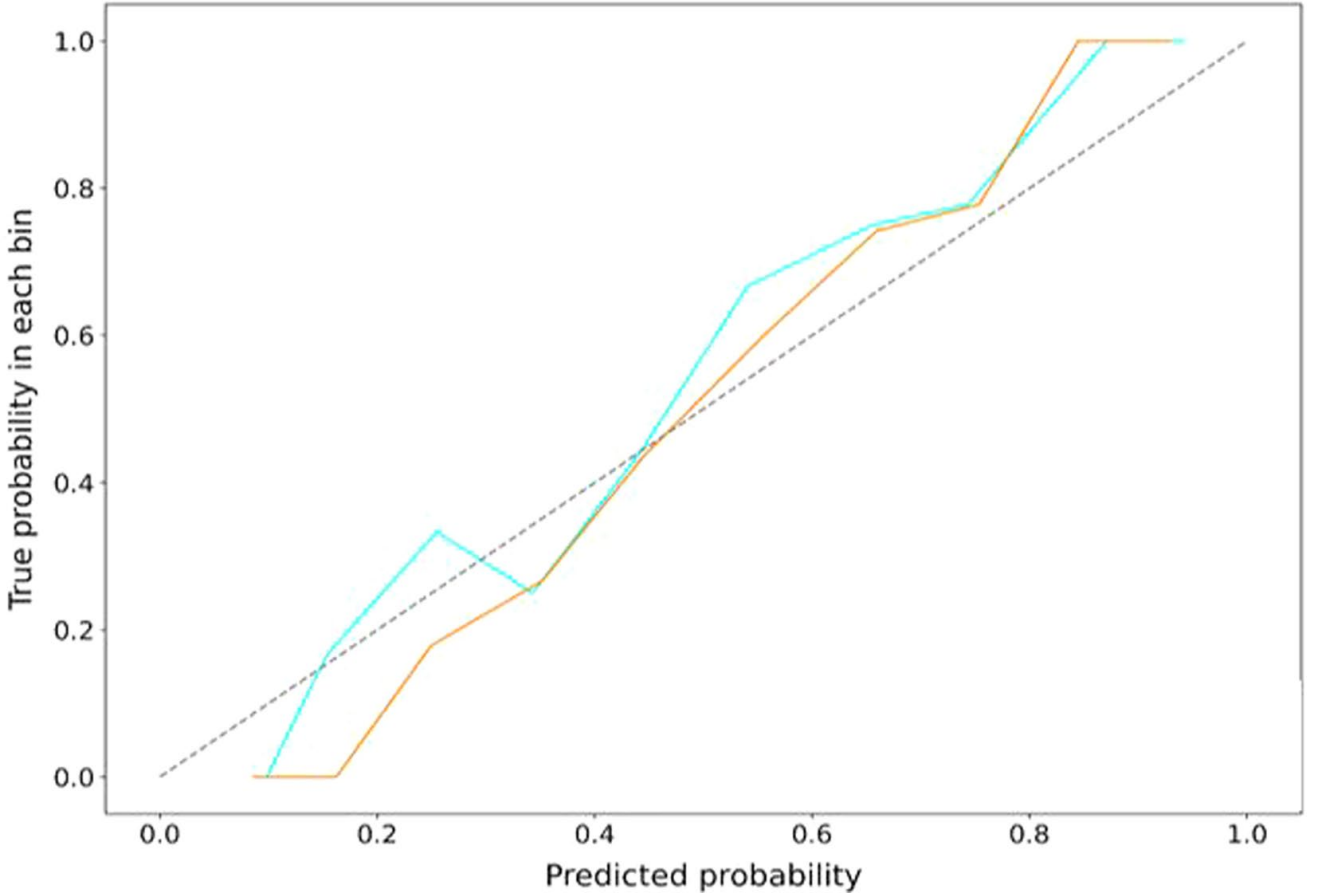
The calibration curves of overall model in the training and test dataset. The closer the calibration curve is to the diagonal line, the higher the calibration degree of the model

The calibration curve demonstrated good fitness of the agreement between the training and test sets of the overall model (Fig. 5). The Hosmer-Lemeshow test showed no significant difference between the predictive probabilities of MACE and the actual probabilities (all *P* > 0.05), which demonstrated good calibration.

### Clinical usefulness

**Fig. 6:**
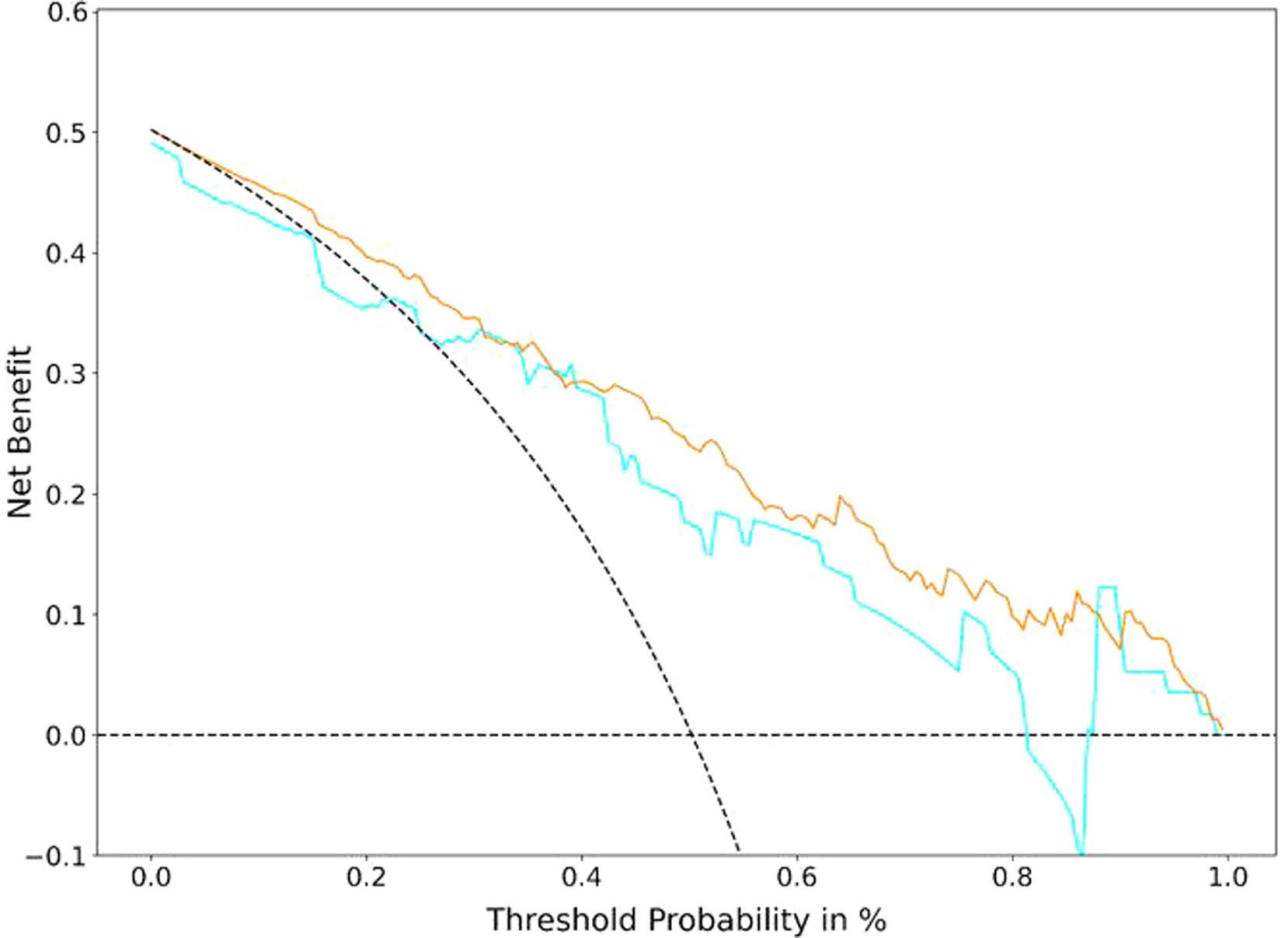
Decision curves of the overall model in the training and test dataset. The decision curve showed that if the threshold probability was between 30 and 80%, using the overall model to predict probability for MACE added more benefit. The *y*-axis indicates the net benefit. The *x*-axis indicates threshold probability. The gray line represents the assumption all patients have MACE. The horizontal line represents the assumption no patients have MACE.

After multivariate logistic regression analysis, the decision curves of the overall model in both the training and test sets are shown in Fig. 6 and were used to determine whether the models provided high net benefit for patients with CAD. The DCA demonstrated that for predicting MACE in patients with CAD, the overall model had an excellent overall net benefit within the majority of reasonable threshold probabilities.

## Discussion

In this study, we built an overall model that comprehensively incorporated clinical risk factors (TG, LDL-C, and hs-CRP) and radiomics features (based on the PCAT surrounding the RCA, LAD, and LCX) to predict MACE within 3 years. The model outperformed the clinical factor-only and radiomics-only models in predicting MACE. Moreover, the integration of the PCAT surrounding the three main coronary arteries revealed higher performance of the PCAT-model than that of the RCA-model, LAD-model, or LCX-model alone. Furthermore, the PCAT surrounding the RCA demonstrated better performance than that surrounding the LAD or LCX.

In patients with CAD, predicting the risk of coronary artery adverse events is more important than assessing the degree of luminal stenosis. The relationship between inflammation and vulnerable plaque is well-documented in the literature [18–20]. Recently, Antonopoulos et al. established a link between PCAT inflammation and CT attenuation in a landmark study [10]. PCAT studies have shown that both pan-coronary and specific lesion inflammation are associated with high-risk lesions [12, 21] and adverse cardiac events [11, 22]. Moreover, the predictive value of perivascular FAI was reported in the CRISP-CT study, which found that although FAI is a strong predictor of all-cause and cardiac mortality, it loses its predictive value in patients whose treatment was initiated with statins and aspirin after CCTA, which suggests that the risk identified by FAI is modifiable [11]. Thus, additional biomarkers are required to detect permanent changes in PCAT composition, which include fibrosis and microvascular remodeling [23, 24]. Radiomics enables the quantification of morphological features that are difficult to discern by the naked eye [25, 26].

Oikonomou et al. [27] were the first to use a radiotranscriptomic method to quantify a CT radiomics profile of adipose composition, which was subsequently linked to the expression of genes that characterize inflammation, fibrosis, and vascularity. They put forward a new artificial intelligence-powered imaging biomarker, the Fat Rradiomics Profile (FRP), and showed that FRP significantly improved the risk prediction of MACE beyond convention risk stratification. Our results are in line with these findings, and the number of adverse events in our study was relatively large and we comprehensively considered the radiomics features of adipose tissue surrounding the three main vessels. Shang et al. recently reported a CCTA-based radiomics characterization of PCAT surrounding target lesions and significant plaque predictor to predict acute coronary syndrome [28]. In contrast, we automatically segmented and extracted radiomics features of PCAT surrounding the three proximal coronary arteries, and the clinical endpoint included patients who died that could respond more comprehensively to MACE. In a prospective case-control study, Lin et al. reported that patients with acute MI exhibit a PCAT radiomics phenotype that is distinct from that of patients with stable or no CAD [16]; however, only RCA was considered and MACE was not predicted. Our study focused on PCAT measurements of the three coronary arteries and indicated that RCA has the highest prediction ability for MACE and LCX has the poorest. These discrepant findings may be due to differences in anatomy and surrounding tissues. The proximal RCA has an abundance of surrounding adipose tissue and an absence of confounding non-fatty structures, such as major side branches, coronary veins, and the myocardium [10, 29]. Compared with RCA and LAD, the anatomical variation and distortion of LCX are relatively larger, and it is surrounded by less adipose tissue. Furthermore, the predictive efficiency was further improved after integrating the RCA, LAD, and LCX. This may be because the radiomics features of PCAT surrounding the LAD and LCX provide additional information.

In our study, radiomics features of PCAT were automatically measured in CCTA images using dedicated software, without the need for extra image acquisition or radiation exposure. Moreover, the clinical risk factors are those that are readily available and routinely collected from medical history.

Our study has several limitations. First, our retrospective study was a preliminary study that was performed in a single center using the same CT scanner with a relatively small sample size. It is well-established that scanners and protocols vary among different hospitals. Several studies have shown that imaging parameters, reconstruction settings, and segmentation algorithms affect radiomics signature of lesions. Thus, the generalizability of the model may be affected [30–32]. Therefore, further studies in a larger population with samples acquired on different scanners are warranted to verify the reproducibility and robustness of our predictive models. Second, our study was a case-control study; causal inferences are limited. In addition, this is a ‘hypothesis generating’ research; thus, our results and conclusions need to be validated in a real-world setting.

In conclusion, the CCTA-based radiomics model of PCAT, integrating the three major proximal coronary arteries, was superior to that of RCA, LAD, and LCX alone in predicting MACE within 3 years. Comprehensively incorporating radiomics features and clinical factors contribute to improve the risk stratification of patients with CAD. Adding radiomics analysis of PCAT to the clinical cardiovascular risk factors can provide incremental prognostic value without any additional financial burden or radiation dose to patients.

### Electronic supplementary material

Below is the link to the electronic supplementary material.

Supplementary Material 1

## Data Availability

The datasets analyzed in this study are available from the corresponding author on reasonable request.

## Abbreviations

MACE: Major adverse cardiovascular event
CCTA: Coronary computed tomography angiography
PCAT: Pericoronary adipose tissue
GBDT: Gradient boosting decision tree
RCA: Right coronary artery
LAD: Left anterior descending artery
LCX: Left circumflex coronary artery
CAD: Coronary artery disease
MI: Myocardial infarction
ACS: Acute coronary syndrome
ICA: Invasive coronary angiography
PCI: Percutaneous coronary intervention
CABG: Coronary artery bypass grafting
FAI: Fat attenuation index
FRP: Fat radiomics profile
CI: Confidence interval
BMI: Body mass index
DCA: Decision curve analysis
