# Supplementary material for: Predicting major adverse cardiovascular events within 3 years by optimization of radiomics model derived from pericoronary adipose tissue on coronary computed tomography angiography: a case-control study
Source: BMC Med Imaging. 2024 May 21;24:117. doi: 10.1186/s12880-024-01295-4 (PMC11110286; doi:10.1186/s12880-024-01295-4)
Supplement: Supplementary file 1 — Supplementary Material 1 [file 12880_2024_1295_MOESM1_ESM.docx]

**Supplementary materials**

**Materials and methods**

**The radiomics features of PCAT**

**Table S1.** List of radiomic features

| **Classification** | **Features (n=95)** |
| --- | --- |
|  | Mesh Volume/mm3, fat attenuation index (FAI) |
| First Order Statistics | Energy, total energy, entropy, minimum, 10th percentile, 90th percentile, maximum, mean, median, interquartile range, range, Mean Absolute Deviation (MAD) , rMAD, Root mean squared, skewness, kurtosis, variance, uniformity |
| Gray Level Co-occurrence Matrix (GLCM) | Autocorrelation, joint average, cluster prominence, cluster tendency, cluster shade, contrast1, correlation, difference average, difference entropy, difference variance, joint energy, joint entropy, informational measure of correlation 1, informational measure of correlation 2, inverse difference moment, inverse difference moment normalized, inverse difference, inverse difference normalized, MCC, inverse variance, maximum probability, sum average, sum entropy, sum of squares |
| Gray Level Size Zone Matrix (GLSZM) | Small area emphasis, large area emphasis, gray level non-uniformity, gray level non-uniformity normalized, size zone non-uniformity, size zone non-uniformity normalized, zone percentage, gray level variance, zone variance, zone entropy, low gray level zone emphasis, high gray level zone emphasis, small area low gray level emphasis, small area high gray level emphasis, large area low gray level emphasis, large area high gray level emphasis |
| Gray Level Run Length Matrix (GLRLM) | Short run emphasis, Long run emphasis, gray level non-uniformity, gray level non-uniformity normalized, run length non-uniformity, run length non-uniformity normalized, run percentage, gray level variance, run variance, run entropy, low gray level run emphasis, high gray level run emphasis, short run low gray level emphasis, short run high gray level emphasis, long run low gray level emphasis, long run high gray level run emphasis |
| Neigbouring Gray Tone Difference Matrix (NGTDM) | Coarseness, contrast2, busyness, complexity, strength |
| Gray Level Dependence Matrix (GLDM) | Small dependence emphasis, Large dependence emphasis, gray level non-uniformity, dependence non-uniformity, dependence non-uniformity normalized, gray level variance, dependence variance, dependence entropy, low gray level emphasis, high gray level emphasis, small dependence low gray level emphasis, small dependence high gray level emphasis, large dependence low gray level emphasis, large dependence high gray level emphasis |

**Results**

After the LASSO analysis, 15, 9, 7, and 15 features remained for RCA, LAD, LCX, and PCAT respectively (Figure.1).


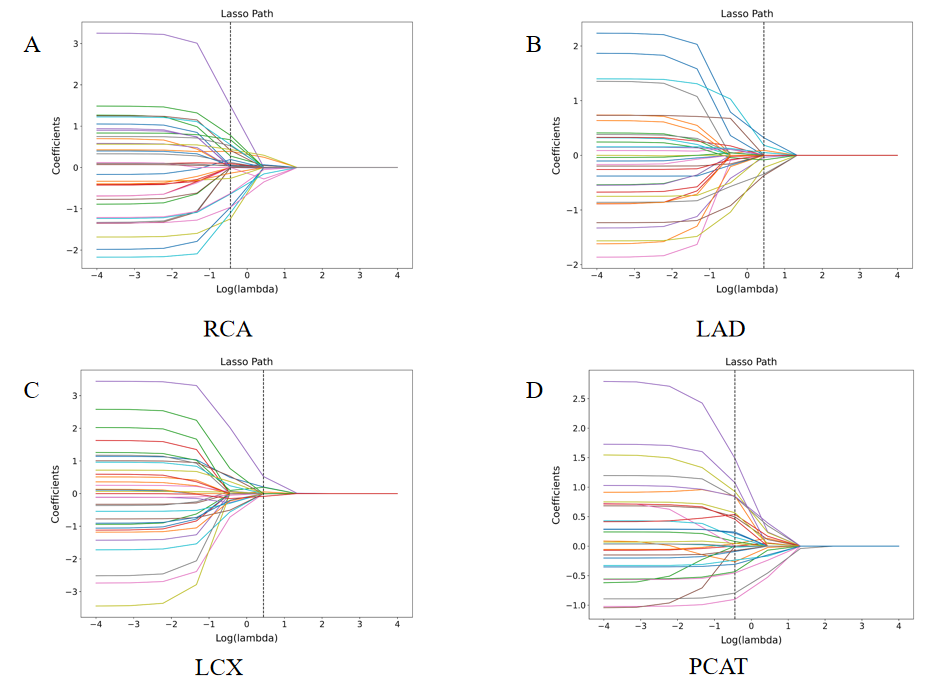


**Table S2.** The performance metrics of the different modeling techniques.

The performance of RCA-model in different machine learning algorithms.

| ML algorithm | Accuracy | |  | AUC (95%CI) | |  | Sensitivity | |  | Specificity | |  | PPV | |  | NPV | |
| --- | --- | --- | --- | --- | --- | --- | --- | --- | --- | --- | --- | --- | --- | --- | --- | --- | --- |
|  | Train | Test |  | Train | Test |  | Train | Test |  | Train | Test |  | Train | Test |  | Train | Test |
| LR | 0.671 | 0.649 |  | 0.708 (0.641, 0.775) | 0.666 (0.518, 0.813) |  | 0.531 | 0.500 |  | 0.813 | 0.793 |  | 0.741 | 0.700 |  | 0.632 | 0.622 |
| SVM | 0.662 | 0.684 |  | 0.706 (0.638, 0.773) | 0.675 (0.531, 0.820) |  | 0.584 | 0.571 |  | 0.741 | 0.793 |  | 0.695 | 0.727 |  | 0.639 | 0.657 |
| SGD | 0.671 | 0.667 |  | 0.699 (0.631, 0.767) | 0.669 (0.521, 0.817) |  | 0.637 | 0.500 |  | 0.705 | 0.828 |  | 0.686 | 0.737 |  | 0.658 | 0.632 |
| LinearSVC | 0.684 | 0.649 |  | 0.705 (0.638, 0.773) | 0.674 (0.529, 0.819) |  | 0.655 | 0.571 |  | 0.714 | 0.724 |  | 0.698 | 0.667 |  | 0.672 | 0.636 |

The performance of LAD-model in different machine learning algorithms.

| ML algorithm | Accuracy | |  | AUC (95%CI) | |  | Sensitivity | |  | Specificity | |  | PPV | |  | NPV | |
| --- | --- | --- | --- | --- | --- | --- | --- | --- | --- | --- | --- | --- | --- | --- | --- | --- | --- |
|  | Train | Test |  | Train | Test |  | Train | Test |  | Train | Test |  | Train | Test |  | Train | Test |
| LR | 0.636 | 0.579 |  | 0.674 (0.604, 0.743) | 0.638 (0.489, 0.787) |  | 0.699 | 0.750 |  | 0.571 | 0.414 |  | 0.622 | 0.553 |  | 0.653 | 0.632 |
| SVM | 0.662 | 0.649 |  | 0.679 (0.609, 0.748) | 0.664 (0.517, 0.811) |  | 0.699 | 0.785 |  | 0.625 | 0.517 |  | 0.653 | 0.611 |  | 0.673 | 0.714 |
| SGD | 0.640 | 0.632 |  | 0.659 (0.588, 0.730) | 0.680 (0.539, 0.821) |  | 0.566 | 0.571 |  | 0.714 | 0.689 |  | 0.667 | 0.640 |  | 0.620 | 0.625 |
| LinearSVC | 0.662 | 0.632 |  | 0.677 (0.607, 0.747) | 0.660 (0.514, 0.807) |  | 0.681 | 0.750 |  | 0.643 | 0.517 |  | 0.658 | 0.600 |  | 0.667 | 0.682 |

The performance of LCX-model in different machine learning algorithms.

| ML algorithm | Accuracy | |  | AUC (95%CI) | |  | Sensitivity | |  | Specificity | |  | PPV | |  | NPV | |
| --- | --- | --- | --- | --- | --- | --- | --- | --- | --- | --- | --- | --- | --- | --- | --- | --- | --- |
|  | Train | Test |  | Train | Test |  | Train | Test |  | Train | Test |  | Train | Test |  | Train | Test |
| LR | 0.640 | 0.561 |  | 0.649 (0.577, 0.720) | 0.613 (0.465, 0.762) |  | 0.620 | 0.571 |  | 0.661 | 0.552 |  | 0.648 | 0.552 |  | 0.633 | 0.571 |
| SVM | 0.636 | 0.614 |  | 0.651 (0.580, 0.723) | 0.623 (0.474, 0.773) |  | 0.664 | 0.643 |  | 0.607 | 0.586 |  | 0.630 | 0.600 |  | 0.642 | 0.630 |
| SGD | 0.618 | 0.632 |  | 0.637 (0.565, 0.709) | 0.589 (0.435, 0.742) |  | 0.593 | 0.571 |  | 0.643 | 0.690 |  | 0.626 | 0.640 |  | 0.610 | 0.625 |
| LinearSVC | 0.631 | 0.597 |  | 0.656 (0.585, 0.727) | 0.622 (0.473, 0.771) |  | 0.779 | 0.750 |  | 0.482 | 0.448 |  | 0.603 | 0.568 |  | 0.684 | 0.650 |

The performance of PCAT-model in different machine learning algorithms.

| ML algorithm | Accuracy | |  | AUC (95%CI) | |  | Sensitivity | |  | Specificity | |  | PPV | |  | NPV | |
| --- | --- | --- | --- | --- | --- | --- | --- | --- | --- | --- | --- | --- | --- | --- | --- | --- | --- |
|  | Train | Test |  | Train | Test |  | Train | Test |  | Train | Test |  | Train | Test |  | Train | Test |
| LR | 0.729 | 0.632 |  | 0.775 (0.715, 0.834) | 0.703 (0.564, 0.843) |  | 0.885 | 0.786 |  | 0.571 | 0.483 |  | 0.676 | 0.595 |  | 0.831 | 0.700 |
| SVM | 0.702 | 0.649 |  | 0.764 (0.703, 0.825) | 0.723 (0.589, 0.857) |  | 0.797 | 0.714 |  | 0.607 | 0.586 |  | 0.672 | 0.625 |  | 0.747 | 0.680 |
| SGD | 0.707 | 0.614 |  | 0.767 (0.706, 0.828) | 0.692 (0.551, 0.833) |  | 0.920 | 0.821 |  | 0.491 | 0.414 |  | 0.646 | 0.575 |  | 0.859 | 0.706 |
| LinearSVC | 0.729 | 0.632 |  | 0.775 (0.716, 0.835) | 0.707 (0.568, 0.846) |  | 0.876 | 0.786 |  | 0.580 | 0.483 |  | 0.678 | 0.595 |  | 0.823 | 0.700 |

The performance of Cli-model in different machine learning algorithms.

| ML algorithm | Accuracy | |  | AUC (95%CI) | |  | Sensitivity | |  | Specificity | |  | PPV | |  | NPV | |
| --- | --- | --- | --- | --- | --- | --- | --- | --- | --- | --- | --- | --- | --- | --- | --- | --- | --- |
|  | Train | Test |  | Train | Test |  | Train | Test |  | Train | Test |  | Train | Test |  | Train | Test |
| LR | 0.702 | 0.684 |  | 0.726 (0.660, 0.792) | 0.683 (0.535, 0.832) |  | 0.522 | 0.500 |  | 0.884 | 0.862 |  | 0.819 | 0.778 |  | 0.647 | 0.641 |
| SVM | 0.702 | 0.667 |  | 0.752 (0.689, 0.815) | 0.706 (0.564, 0.847) |  | 0.460 | 0.464 |  | 0.946 | 0.862 |  | 0.897 | 0.765 |  | 0.635 | 0.625 |
| SGD | 0.702 | 0.719 |  | 0.726 (0.660, 0.792) | 0.692 (0.546, 0.838) |  | 0.496 | 0.500 |  | 0.911 | 0.931 |  | 0.849 | 0.875 |  | 0.642 | 0.659 |
| LinearSVC | 0.707 | 0.684 |  | 0.725 (0.659, 0.791) | 0.685 (0.538, 0.832) |  | 0.522 | 0.500 |  | 0.893 | 0.862 |  | 0.831 | 0.778 |  | 0.649 | 0.641 |

The performance of Overall-model in different machine learning algorithms.

| ML algorithm | Accuracy | |  | AUC (95%CI) | |  | Sensitivity | |  | Specificity | |  | PPV | |  | NPV | |
| --- | --- | --- | --- | --- | --- | --- | --- | --- | --- | --- | --- | --- | --- | --- | --- | --- | --- |
|  | Train | Test |  | Train | Test |  | Train | Test |  | Train | Test |  | Train | Test |  | Train | Test |
| LR | 0.756 | 0.702 |  | 0.827 (0.774, 0.879) | 0.791 (0.671, 0.911) |  | 0.779 | 0.607 |  | 0.732 | 0.793 |  | 0.746 | 0.739 |  | 0.766 | 0.677 |
| SVM | 0.760 | 0.719 |  | 0.828 (0.776, 0.881) | 0.797 (0.679, 0.915) |  | 0.770 | 0.643 |  | 0.750 | 0.793 |  | 0.757 | 0.750 |  | 0.764 | 0.697 |
| SGD | 0.760 | 0.719 |  | 0.828 (0.776, 0.881) | 0.799 (0.682, 0.916) |  | 0.779 | 0.679 |  | 0.741 | 0.759 |  | 0.752 | 0.731 |  | 0.769 | 0.710 |
| LinearSVC | 0.760 | 0.702 |  | 0.827 (0.775, 0.880) | 0.796 (0.677, 0.914) |  | 0.761 | 0.643 |  | 0.760 | 0.759 |  | 0.761 | 0.720 |  | 0.759 | 0.688 |

**Table S3.** ROC values of LAD-model, LCX-model and RCA-model at different tube voltages.

| ROC | KVp | | p value |
| --- | --- | --- | --- |
|  | 100 | 120 |  |
| LAD-model | 0.721 | 0.680 | 0.709 |
| LCX-model | 0.545 | 0.667 | 0.328 |
| RCA-model | 0.597 | 0.722 | 0.317 |
